# Breast cancer incidence following low-dose rate environmental exposure: Techa River Cohort, 1956–2004

**DOI:** 10.1038/sj.bjc.6604775

**Published:** 2008-11-11

**Authors:** E Ostroumova, D L Preston, E Ron, L Krestinina, F G Davis, M Kossenko, A Akleyev

**Affiliations:** 1 Radiation Epidemiology Branch, Division of Cancer Epidemiology and Genetics, National Cancer Institute, NIH, MS 7238, 6120 Executive Boulevard, Bethesda, MD 20892-7238, USA; 2 Laboratory of Epidemiology, Urals Research Center for Radiation Medicine, 68-a Vorovsky St, Chelyabinsk 454076, Russia; 3 Hirosoft International Corporation, 1335 H St, Eureka, CA 95501, USA; 4 Division of Epidemiology and Biostatistics, School of Public Health, University of Illinois at Chicago, 877 SPHPI, M/C 923, 1603 W Taylor St, Chicago, IL 60612, USA

**Keywords:** breast cancer, incidence, ionising radiation, low-dose rate exposure, Techa River

## Abstract

In the 1950s, the Mayak nuclear weapons facility in Russia discharged liquid radioactive wastes into the Techa River causing exposure of riverside residents to protracted low-to-moderate doses of radiation. Almost 10 000 women received estimated doses to the stomach of up to 0.47 Gray (Gy) (mean dose=0.04 Gy) from external *γ*-exposure and ^137^Cs incorporation. We have been following this population for cancer incidence and mortality and as in the general Russian population, we found a significant temporal trend of breast cancer incidence. A significant linear radiation dose–response relationship was observed (*P*=0.01) with an estimated excess relative risk per Gray (ERR/Gy) of 5.00 (95% confidence interval (CI), 0.80, 12.76). We estimated that approximately 12% of the 109 observed cases could be attributed to radiation.

The female breast is recognised as one of the most radiation-sensitive organs and exposure to ionising radiation appears to play a role in both the initiation and promotion of breast cancer (UNSCEAR, 2000). Increased risk of breast cancer was reported in women exposed to multiple X-ray examinations (Boice *et al*, 1991; Doody *et al*, 2000), radiotherapy (Hildreth *et al*, 1989; Mattsson *et al*, 1993; Lundell *et al*, 1999) and radiation from the atomic bombings in Japan (Land *et al*, 2003; Preston *et al*, 2007). The atomic bombings and fractionated medical X-radiation are characterised by acute, high-dose rate *γ*-radiation. In a pooled analysis of several cohorts, an excess relative risk (ERR) per Gray (Gy) of 0.86 (95% confidence interval (CI), 0.7, 1.04) was reported with individual study risks ranging from 0.06 to 1.94 (Preston *et al*, 2002).

In contrast, risks from exposure to chronic low-dose rate radiation have rarely been quantified due to a lack of adequate dose estimates. Associations between cancer risks from low-dose occupational radiation exposure (Wang *et al*, 2002; Doody *et al*, 2006) or protracted low-to-moderate dose environmental radiation exposure (Bauer *et al*, 2005; Pukkala *et al*, 2006) have, however, been reported.

In this paper, we report on the breast cancer experience among women who were exposed to protracted low-dose rate external and internal radiation from discharges of radioactive waste into the Techa-Iset river system from the Mayak nuclear weapons facility in the Southern Urals region of Russia. These releases primarily occurred between 1950 and 1960, affecting tens of thousands of inhabitants of riverside villages. The study was reviewed and approved by the institutional review board at the Urals Research Center for Radiation Medicine (URCRM).

## Materials and methods

### Study cohort

The Techa River Incidence cohort (TRIC) consists of 18 382 individuals born before 1950, who lived in villages on the Techa River in Chelyabinsk Oblast at some time between January 1950 and December 1960. The current analyses are based on the 9908 female cohort members with follow-up from 1956–2004. The study cohort and follow-up period were defined on the basis of the cancer incidence data availability.

The discharge of radioactive wastes into the Techa-Iset river system was the source of a substantial radiation exposure. The total volume of radioactive wastes discharged by the Mayak facility amounted to 76 million m^3^ with a total activity of 10^17^ Bq (2.75 million Ci) for *β*-emitters (Akleyev and Lyubchansky, 1994). About 95% of the total activity was released into the Techa River from March 1950 through November 1951. The primary radionuclides released were ^137^Cs and the bone-seeking isotopes of strontium (^89^Sr and ^90^Sr), which accounted for 12.2 and 20.4% of the total discharge, respectively (Degteva *et al*, 1994).

Residents of the riverside areas received both external and internal radiation exposures. External exposures to *γ*-radiation were primarily from ^137^Cs, with some additional exposure from ^106^Ru, ^95^Zr. Internal exposures largely resulted from use of river water contaminated with ^137^Cs, ^89^Sr, and ^90^Sr for drinking, cooking and other domestic needs (Degteva *et al*, 1994). Details of the Techa River Dosimetry System (TRDS-2000) used to reconstruct external and internal doses are published elsewhere (Degteva *et al*, 2000a, 2000b; Jacob *et al*, 2003). Exposure of soft tissues, including breast, was largely from external *γ*-radiation and internal *γ*-radiation from ^137^Cs distributed uniformly through the body. In the absence of individual breast dose estimates, individualised estimates of doses to the stomach are used as an approximation of dose to breast. The TRDS-2000 provides annual dose estimates based primarily on age- and village-specific group means that were individualised to account for the periods of residence in contaminated villages. The maximum estimated dose to the stomach was 0.47 Gy (mean=0.04 Gy; median=0.01 Gy).

Considerable migration among study cohort members because of compulsory re-settlement from highly contaminated to uncontaminated villages, and migration of younger cohort members primarily for educational or job opportunities presented a challenge in follow-up and therefore most work has focused on mortality data (Kossenko *et al*, 2005; Krestinina *et al*, 2005). Information on date and place of death, current residence and the date and place of migration of cohort members was obtained through periodic queries to the Address Bureaus of Chelyabinsk and Kurgan Oblasts. Because of the difficulties in obtaining cancer incidence data, we limited follow-up to the five rayons (districts) in Chelyabinsk Oblast where many of the exposed subjects resided and to Chelyabinsk city. These six geographic areas are considered the cancer incidence study catchment area.

Reporting of newly diagnosed malignancies to the Regional Oncology Dispensaries using notification forms became mandatory in the former Soviet Union in 1956. URCRM staff have systematically collected copies of cancer notification forms for residents of the study catchment area. As the probability of identifying a cancer case depended on a cohort member living in the study catchment area, only breast cancers diagnosed while the patient lived in the catchment area are included in the analysis. Cohort members are treated as lost to follow-up when they migrate from the catchment area (Kossenko *et al*, 2005; Krestinina *et al*, 2007).

Basic personal identifiers (last name, first name and father's first name, birth date, place of birth) are used for matching cancer case notifications to the TRIC. Copies of death certificates for deceased cohort members are obtained from the Regional Vital Statistics Registrar's Office archives. Additional information on cohort members who developed breast cancer is obtained from their medical charts stored at the URCRM clinical department.

### Statistical methods

Analyses were based on a detailed classification of person-years and case counts stratified by ethnic group (Slavs and Tartars/Bashkirs), attained age (5-year groups through age 79 and 80 plus), follow-up period (5-year periods from 1956 through 2004), age at first exposure (10-year groups through age 59 and 60 plus), time of study entry (before and after 1.1.1953), number of live births (nulliparous, 1–2, ⩾3 children and unknown), age at first pregnancy (<25, ⩾25 years and unknown), and stomach dose (in Gy) with 5-year latency period, that is, dose received in ⩽5 years prior to cancer diagnosis was not considered in assessing breast cancer risk. The first 6 years of follow-up (1950–1955) were excluded from analysis, because cancer incidence data were not available. Women contributed person-years only when they were known to reside in the catchment area, as this reflected when a woman was at risk of having a breast cancer identified. Person-year accumulation began on 1 January 1956 or from the initial date of residence in a contaminated village after that date. The termination date for counting person-years was the date of death, or the earliest of the following: date of last known residence in the catchment area, or of breast cancer diagnosis, or 31 December 2004. Person-year computations and risk estimation were carried out using Epicure software (Preston *et al*, 1993).

Statistical analyses and calculation of risk coefficients were based on both external and internal comparisons. For the external comparisons, the number of expected cases was calculated using both general and rural Russian female population age- and calendar-period-specific incidence rates (Tserkovnyi *et al*, 1975; Napalkov *et al*, 1982; Merabishvili *et al*, 1984; Dvoirin *et al*, 1991; Dvoirin and Aksel, 1992; Chissov and Starinskii, 2000; Chissov *et al*, 2006). The ratio between observed and expected number of cancers, the standardised incidence ratio (SIR) was calculated with 95% CIs assuming a Poisson distribution.

Breast cancer incidence rates in the cohort were analysed using simple parametric excess relative risk (ERR) models. The basic ERR model for age-specific breast cancer incidence rates was λ(*a, d, z*)=λ_*0*_(*a, z*_*0*_)(*1*+ρ(*d*)ε(*z*_*1*_)) where *a* is age at diagnosis, *d* is stomach dose in Gy, *z*_*0*_ represents factors (such as birth cohort, parity, age at first full-term pregnancy, ethnicity or date of arrival on the contaminated area) that could modify the baseline rates (*λ*_*0*_), and *z*_*1*_ represents factors that could modify the ERR. For analyses based on external comparisons the baseline rates were taken to be equal to the *θR*_*pop*_(a,y), where R_pop_(*a,y*) is the population rate for age *a* in year *y,* and *θ* is the estimated baseline SIR. For analyses based on internal comparisons, a log linear model was used to estimate baseline (zero-dose) risk with the rates to be proportional to age to a power that changes once women reach the age of 50 years. Ethnicity, number of live births, time of study entry and birth cohort were considered as potential modifiers of the baseline rates.

The dose–response function was generally taken as a linear function of dose (*ρ*_*1*_*d)*. Tests for non-linearity in the dose–response were based on comparison of the linear and linear-quadratic dose–response models (*ρ*_*1*_*d*+ρ_*2*_*d*^*2*^). Because of the small number of observed and radiation-associated cases in this cohort, there is little power to assess effect modification. The primary analysis was based on a dose–response model without effect modification. However, we evaluated several factors that might modify the association between radiation and breast cancer, namely, age at exposure, ethnicity and age at diagnosis.

Parameter estimates were obtained using Poisson regression maximum likelihood analyses of rates in the detailed rate tables described above. Significance tests and CIs were determined directly from the likelihood. All *P*-values refer to two-sided tests.

## Results

Over 37 years (1956–2004), 109 breast cancers were diagnosed among TRIC members, of which 79% were histologically confirmed, 16% by X-ray and clinical examinations, and 5% solely from death certificates.

The majority of cohort members reported Slavic ethnicity (67%), 42% of women were less than 20 years old at the start of exposure, and 79% lived along the river during the period of maximum releases (i.e., 1950–1952). At the end of follow-up 19% of the cohort were urban residents. The estimated stomach doses were below 5 mGy for 30% of the cohort members, whereas 16% had a dose of 50 mGy and over.

Table 1 summarises the follow-up status of the study cohort. The 9908 study subjects have a total of 270 289 person-years of follow-up in the catchment area during the study period. At the end of the follow-up period, 2115 (21%) cohort members were known to have migrated from the catchment area. Owing to lack of complete cancer incidence data outside the catchment area, as well as the differences in follow-up within and outside this area (vital status unknown for 37% of migrants *vs* 8% of non-migrants; cause of death unknown for 41% of deceased outside *vs* 13% of deceased within the catchment area), the analyses were limited to the periods in which cohort members were known to reside in the catchment area.

**Figure 1:**
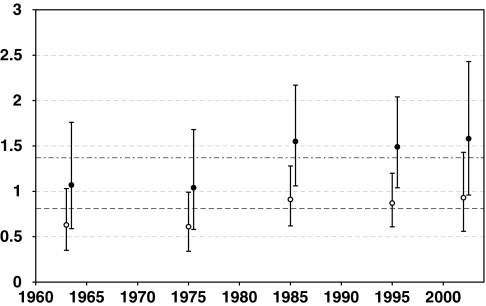
Standardised Incidence Ratios (SIRs) and 95% confidence intervals (CIs) for breast cancer for the Techa River female population compared with Russian general (open circles) and rural (black circles) female populations by follow-up period. Lines represent overall SIR estimate for the Techa River female population compared with Russian rural (upper, dot-dashed line) and Russian general (lower, dashed line) female populations. *x* axis – Follow-up year; *y* axis – SIR.

Table 2 shows crude breast cancer incidence rates and adjusted relative risks by follow-up period. The crude rates increased with follow-up period primarily due to the aging of the cohort. The increase in baseline incidence rates prior to and after 50 years of age was proportional to the 6.8 and 1.1 power of age, respectively. After adjusting for attained age, we found a marked birth cohort effect (*P*<0.001) with rates increasing on average by 50% (95% CI: 28; 87%) per decade increase of birth year. Without allowing for possible radiation dose effects, the number of cases was 37% more than predicted national rates for rural areas (109 observed *vs* 79.6 expected, SIR=1.37; 95% CI: 1.13; 1.65). However, when compared with general female population rates the observed number of cases was 19% lower than expected (109 *vs* 135.3, SIR=0.81; 95% CI: 0.67; 0.97). Allowing for a linear dose–response, the baseline SIRs were somewhat lower, with estimates of 1.26 (rural) (95% CI: 1.01; 1.58) and 0.74 (general) (95% CI: 0.59; 0.93), respectively. Breast cancer SIRs based on Russian general and rural female population rates by follow-up period are presented in Figure 1.

Table 3 summarises relative risk estimates for selected non-radiation risk factors. Background breast cancer risk among Tartar and Bashkir was 34% lower than Slavic women (*P*=0.06). A significantly higher risk was found among women who arrived on the Techa River between 1953 and 1960 (i.e., late entrants) compared with those who lived in the area during the period of maximal radioactive contamination. The highest breast cancer risk was found in nulliparous women compared with women who had three or more children (*P*=0.008). Among parous women, there was no difference in risk by age at first birth (*P*=0.43).

A significant (*P*=0.01) linear dose–response relationship was observed for breast cancer with little evidence of non-linearity (*P*>0.5). The estimated ERR/Gy was 4.99 (95% CI: 0.80; 12.76). Table 4 summarises the distribution of cases, person-years and fitted values in 5-year lagged cumulative dose categories.

Under a linear dose–response model, it was estimated that 12.4% (95% CI: 2.3; 25.5%) of the breast cancers observed were attributable to radiation exposure. The small number of breast cancers limited the ability to detect effect modification. Ethnicity, age at diagnosis, age at exposure and time since exposure did not significantly modify the breast cancer radiation risk estimate; however, women who were under age 10 at first exposure appeared to have the highest risk (data not shown).

## Discussion

This is the first report describing breast cancer incidence patterns and radiation dose relations in a subgroup of the Techa River cohort. The main result from our study was a significant linear relationship between breast cancer incidence and radiation dose. This finding adds to the limited data on the carcinogenic effects from protracted environmental ionising radiation in the low-to-medium dose range.

As neither Techa River residents nor health care providers were aware of dose levels and given the legal requirement for newly diagnosed malignancies to be reported to the Regional Oncology Dispensary, the likelihood of differential reporting for patients with high and low exposure is small. The power of the study is decreased by the reduction in person-years of follow-up because of relatively high migration from the study catchment area. Because of the nature of the address and cancer registration systems it is not possible to obtain complete information on cancer incidence, vital status or cause of death for cohort members living outside the study catchment area. For cohort members living in the study catchment area, vital status at the end of follow-up was known for 92% and among deceased subjects cause of death was ascertained for 87% (Table 1). However, vital status at the end of follow-up was known for only 63% of cohort members who had left the catchment area and cause of death was known for only 60% of the identified. Therefore, analyses were limited to periods in which subjects were known to reside in the study catchment area. The loss to follow-up reduces the study's statistical power to detect a dose–response and assess effect modification. It could introduce some bias, but as there is no indication that the loss to follow-up is dose-related, bias is unlikely. In our study, 79% of breast cancers had histological confirmation and 5% were identified only through death certificates. This is comparable with the respective indices in Eastern Europe cancer registries (IARC, 1992).

We found a significant increase of breast cancer background risk with attained age, nulliparity, and follow-up time, as in the general Russian population and worldwide (Remennick, 1989; Zaridze and Basieva, 1990; IARC, 1993). Although national data are limited, the striking birth cohort effect in our study appears to be consistent with that in the Russian Federation as a whole during this period (Tserkovnyi *et al*, 1975; Napalkov *et al*, 1982; Merabishvili *et al*, 1984; Dvoirin *et al*, 1991; Dvoirin and Aksel, 1992; Chissov and Starinskii, 2000; Chissov *et al*, 2006).

The failure to find a significant effect of age at first full-term pregnancy can be attributed to the fact that 97% of women in our study were under 30 years of age at the time of their first full-term pregnancy. After adjustment for the number of live births, the difference between the two ethnic groups (Slavs *vs* Tartars and Bashkirs) was marginally significant (*P*=0.06) suggesting that this is largely because of different reproductive patterns (Remennick, 1989). The reason for higher background breast cancer risk among late entrants compared with women resident in the Techa area in 1950–1952, is not clear and requires further investigation.

We recognise the possible underascertainment of breast cancers in our cohort because of the retrospective nature of our follow-up, most likely in women 70 years and older. Breast cancer SIR estimates were somewhat higher (SIR=1.37; 95% CI: 1.13; 1.65) using Russian rural rates, and lower (SIR=0.81; 95% CI: 0.67; 0.97) with the general Russian rates were used.

One of the main limitations in our study was the relatively low statistical power for quantifying how the radiation risk varies with age at exposure, age at diagnosis and ethnicity. Although it is significant, the point estimate of the ERR per Gy of 4.99 is quite uncertain with 95% CI, 0.80, 12.76. Although the TRDS-2000 does not provide breast dose estimates, because of the nature of the soft tissue exposures (external *γ*-ray exposures and internal exposure to *γ-*rays from ^137^Cs distributed uniformly throughout the body), the breast and stomach doses should be similar. TRDS-2000 dose estimates should not be considered definitive, as efforts are currently underway to improve the dosimetry system by better characterisation of the radiation source term and using more information to individualise dose estimates (Degteva *et al*, 2006).

On account of the overlapping CIs, our risk estimate, though higher, is comparable with the ERR of 0.86/Gy (95% CI: 0.7; 1.04) found in the pooled analysis of atomic bomb survivors and seven cohorts of medically irradiated women with 1502 breast cancers and 1.8 million person-years of follow-up (Preston *et al*, 2002). Among the cohorts considered in that study, the highest ERR (1.94/Gy with a 95% CI of 1.3–2.8) was observed in the Swedish benign breast disease cohort (mean breast dose=5.8 Gy).

Significantly elevated breast cancer risks were reported for a cohort of 56 436 US radiologic technologists (Doody *et al*, 2006) and among 5443 Chinese female X-ray workers (Wang *et al*, 2002) who experienced daily, small radiation doses over many years. In both of these cohorts the highest relative risks appeared to be among women who were younger at the time of initial exposure. A meta-analysis of breast cancer incidence among female flight attendants suggested a significant increase in risk due to occupational exposure to cosmic radiation (Tokumaru *et al*, 2006). Rafnsson *et al* (2001) also found an increased breast cancer risk among 1690 flight attendants with 26 breast cancers (SIR=1.5, 95% CI: 1.0; 2.4) at the mean annual radiation dose of about 3 mSv. The authors suggested that the doses appeared to be too low to explain the increased risk and stated that adjustment for other occupational hazards (e.g., exposure to chemicals, irregular work hours etc.) was necessary.

Analysis of 61 breast cancers in almost 5000 women exposed to radiation from nuclear testing fallout in Kazakhstan (mean effective dose=634 mSv), provided an ERR of 1.09/Sv (95% CI: −0.05; 15.8) (Bauer *et al*, 2005). A study of breast cancer incidence after the Chernobyl accident found an increased risk among women resident in the most contaminated districts (average cumulative whole body dose of 50 mSv) with an ERR of 1.2 (95% CI, 0.5, 2.3) in Belarus and 0.8 in Ukraine (95% CI: 0.1; 1.9). However, the limitations of ecologic studies require that this finding be interpreted with caution (Pukkala *et al*, 2006).

Our study findings are in agreement with the hypothesis of linearity of radiation dose–response for breast cancer. The point estimate of the ERR per Gy is higher than that reported by others, but given our wide risk CIs, it is consistent with many other studies. Current dose estimates have limitations that could bias ERR estimates (Degteva *et al*, 2006). Planned improvements to the dosimetry system include: (1) use of modified source term based on information on the timing and composition of the Mayak nuclear facility releases: (2) taking account of the precise location of subjects' residence rather than the current use of a representative location for each village (an important factor for external dose); (3) using improved information on an individual's source of drinking water (affecting internal dose); (4) making more direct use of individual whole body count data in estimation of internal doses; (5) taking into account other sources of radiation exposure, such as the 1957 Kyshtym accident, radioactive gaseous aerosol releases from the Mayak nuclear facility, and exposure from radiological medical examinations. Although these changes will reduce uncertainty of individual dose estimates, they are not expected to substantially modify risk estimates.

In summary, an analysis of breast cancer incidence among women in the Techa River Incidence Cohort has shown a significant increase with age at diagnosis, birth cohort and reduction in incidence with increasing parity. A significant linear trend of risk with increasing radiation dose, is based on small numbers and requires cautious interpretation.

## Figures and Tables

**Table 1:** Distribution of vital status in the Techa River Incidence female cohort by residence for subjects with and without breast cancer (follow-up period 1.1.1956–31.12.2004)

**Vital status as of 31.12.2004**	**Cancer incidence catchment area^a^ (%)**	**Outside the catchment area (%)**	**Total (%)**
*Non cases* (*n*=*9777*)			
Alive	2666–34.2	611–28.9	3277–33.1
Dead	4372–56.1	701–33.1	5073–51.2
*Cause known*	*3820–87*	*412–59*	*4232–83*
*Cause unknown*	*552–13*	*289–41*	*841–17*
Current vital status unknown	646–8.3	781–36.9	1427–14.4
			
*Breast cancer cases* (*n*=*131*)			
Alive	41	4	45
Dead	66	18	84
*Cause known*	*62*	*17*	*79*
*Cause unknown*	*4*	*1*	*5*
Current vital status unknown	2	0	2
Total	7793–100	2115–100	9908–100
Person-years	270289	57684	327973

a Cancer incidence catchment area includes four rayons through which Techa River flows (Kaslinsky, Krasnoarmeysky, Kunashaksky and Argayshsky) and Sosnovsky rayon where many exposed residents were resettled and Chelyabinsk city to which many of the cohort members moved.

**Table 2:** Breast cancer incidence rates and relative risks by times of tumour diagnosis in the Techa River Incidence female cohort

**Follow-up period**	**Number of cases**	**Number of person-years**	**Mean age, years**	**Crude rate per 10^5^ PYR (95% CI)**	**RR^a^ (95% CI)**
1956–1969	13	108300	39.4	12.0 (7.0–20.7)	1.0
1970–1979	14	61056	48.9	22.9 (13.6–38.7)	1.3 (0.6–2.9)
1980–1989	30	48847	55.7	61.4 (42.9–87.8)	2.86 (1.5–5.5)
1990–2004	52	52086	63.5	99.8 (76.17–131.0)	3.9 (2.1–7.7)
**1956–2004**	**109**	**270289**	**49.1**	**40.3** (**33.4**–**48.7)**	

a Relative risk adjusted for age at diagnosis, ethnicity, number of children, time of arrival on the contaminated territory (before and after 1953) and 5-year lagged cumulative stomach dose based on linear ERR model.

**Table 3:** Breast cancer incidence risk estimates

**Risk factor**	**Cases *n*=109**	**PYR *n*=270289**	**RR^a^**	**95% CI**
*Ethnicity*				
Slavs	78	165456	1.0	Referent
Tartars & Bashkirs	31	104833	0.7	0.4–1.0
*P* for homogeneity 0.06, d.f.=1				

*Subcohort*				
OTRC^b^	82	221848	1.0	Referent
Late entrants	27	48441	1.7	1.03–2.6
*P* for homogeneity 0.04, d.f.=1				

*Attained age, years:*				
<45	12	110730	0.1	0.1–0.2
45–54	33	52585	1.0	Referent
55–64	34	50453	1.3	0.8–2.2
65+	30	56521	1.7	1.0–3.0
*P* for homogeneity <0.001, d.f.=3				

*Number of children born* c				
3+	36	124060	1.0	Referent
1–2	53	104546	1.8	1.1–2.7
Nulliparous	19	36483	2.2	1.2–3.8
*P* for homogeneity 0.008, d.f.=2				

*Age at first pregnancy* c				
<20 years	8	27123	1.0	Referent
⩾20 years	81	200998	1.3	0.7–3.0
*P* for homogeneity 0.43, d.f.=1				

a Relative risk adjusted for age at diagnosis, ethnicity, number of children, age at first pregnancy, time of arrival on the contaminated territory (before and after 1953), linear birth cohort effect and 5-year lagged cumulative stomach dose based on linear ERR model.

b OTRC=Original Techa River Cohort (resident in the Techa area in 1950–1952).

c One case with unknown information on live birth and age at first pregnancy is excluded from analysis.

**Table 4:** Breast cancer 1956–2004 by stomach dose category

**Dose^a^ (mGy)**	**Person years**	**Cases**	**Background^b^**	**Excess^b^(95% CI)**	**%AR^c^(95% CI)**
<5.0	67631	25	30.7	0.3 (0.04–0.6)	1.2 (0.2–2.4)
5–9.9	75460	37	29.7	1.0 (0.2–2.1)	2.7 (0.5–5.7)
10–24.9	41377	8	10.5	1.1 (0.2–2.2)	13.7 (2.5–27.5)
25–49.9	48453	19	14.4	2.0 (0.4–4.1)	10.5 (2.1–21.6)
50+	37368	20	10.1	9.1 (1.7–18.7)	45.5 (8.5–93.5)
Total	270289	109	95.5	13.5 (2.5–27.8)	12.4 (2.3–25.5)

a 5-year lagged cumulative stomach dose.

b Estimates of the number of background and radiation-associated excess cases based on a linear ERR model after background rates adjustment for effects of age, number of children, time of arrival on the contaminated territory (before and after 1953) and linear birth cohort effect.

c AR–attributable risk estimated as the ratio of the number radiation-associated excess cases over the total number of cases in each stomach dose category.
